# From routine periodontal therapy to Alzheimer's disease early detection: A scoping review

**DOI:** 10.1177/25424823261421629

**Published:** 2026-02-04

**Authors:** Qiang Zhang, Lina Almanie, Yi Ouyang, Zihao Cheng, Hengjia Zhang

**Affiliations:** 1 Department of Neurology, Shenzhen Lansheng Neurology Hospital, Shenzhen, P.R. China; 2 School of Clinical Dentistry, 111994 University of Sheffield, Sheffield, UK; 3 Department of Periodontics, 612609 King Fahd Specialist Hospital, Buraydah, Saudi Arabia; 4 Department of Neurology, 159407 The First Hospital of China Medical University, China Medical University, Shenyang, P.R. China

**Keywords:** Alzheimer's disease, biomarkers, gingival crevicular fluid, granulation tissue, periodontitis

## Abstract

An epidemiological association has been observed between periodontitis and Alzheimer's disease (AD); however, salivary and blood assays often show low specificity. Periodontal tissues and fluids, which are routinely removed and discarded during periodontal treatment, may be collected to offer matrices useful for the early detection of AD. This study aimed to map current preclinical and clinical evidence on biomarkers measured in periodontal tissues and fluids for the early detection of AD and organize them within an AD-specificity pyramid anchored to brain-relevant endpoints. Following PRISMA-ScR (Preferred Reporting Items for Systematic Reviews and Meta-Analyses–Extension for Scoping Reviews) guidance, we searched PubMed, Scopus, and Web of Science (January 1, 2015–August 31, 2025) for preclinical and clinical studies measuring AD-relevant biomarkers in periodontal matrices. The protocol was pre-registered (OSF DOI: 10.17605/OSF.IO/EDVU9; August 20, 2025). Two reviewers extracted the data, and other two independently verified them. The findings were organized using a four-tier AD-specificity pyramid. Results: Fourteen studies met the inclusion criteria. The biomarkers from the included studies were clustered into microbiome features, molecular signals, and genetic/transcriptomic findings. Evidence ranged from Tier-1 contextual inflammation/pathogens to Tier-4 core-pathology adjacency; five studies incorporated clinical/biological anchoring, with cerebrospinal fluid amyloid-β positivity providing the most brain-relevant anchor. Periodontal matrices are practicable, high-signal sources for AD-relevant biomarkers. However, translational validation linking periodontal biomarkers to brain endpoints is needed to assess the feasibility of multi-tier and chairside panels for early AD detection as part of routine periodontal care.

## Introduction

Alzheimer's disease (AD) is a progressive neurodegenerative disorder projected to affect 13.8 million by 2060.^1^ In 2017, severe periodontitis exhibited an age-standardized global prevalence of approximately 9.8%, and from 2011 to 2020, it affected approximately 23.6% of dentate adults.^2,3^ Observational data indicate that severe periodontitis is associated with higher risks of AD dementia and cognitive impairment.^4,5^ A recent meta-analysis involving over 4.2 million participants revealed approximately 45.0% higher odds of cognitive impairment among older adults with periodontitis.^6^ Increasing evidence implicates chronic peripheral neuroinflammation in AD pathogenesis, with oral infection proposed as an upstream driver of brain immune priming; for example, experimental periodontitis models have demonstrated brain inflammatory changes even in the absence of direct invasion of the oral microbiome.^7,8^ Overall, severe periodontitis is increasingly considered as a potential risk factor for AD.^9–11^

Granulation tissue in deep periodontal pockets is routinely removed as part of standard periodontal treatment because leaving inflamed tissue *in situ* can compromise debridement and pocket healing. During usual care, this material is simply discarded, such as when it is removed through suction.^12,13^ Compared with saliva (often low in abundance and demonstrating variable concordance with AD biomarkers), periodontal tissues and fluids provide higher local analyte levels and enable cellular- or spatially resolved assays (e.g., RNA-seq and spatial transcriptomics).^14^ Recent studies have shown that granulation tissue retrieved from surgeries yields large-scale multi-omics data and is clinically tractable during routine care.^15,16^ Evidence directly linking periodontal tissues and fluids to AD biomarkers remains limited; however, such investigations are a rapidly emerging research focus.^17^

One central limitation is that generic inflammatory or bacterial readouts lack specificity for early AD detection.^18,19^ General inflammatory biomarkers and pathogens, including IL-1β, IL-6, TNF-α, MCP-1, and periodontal pathogens, are responsive to periodontal inflammation but are non-specific to AD.^20,21^ In periodontal fluids, elevated YKL-40 in gingival crevicular fluid or serum largely reflects periodontal inflammation and tissue breakdown.^22^ FITM3–γ-secretase signaling and the NLRP3 inflammasome lie proximal to the amyloidogenic and neuroinflammatory cascades; however, there is limited periodontal/oral-matrix evidence linking these pathways to hard AD endpoints, such as clinical anchoring, A/T/N (amyloid-β, tau, and neurodegeneration) status, or cognitive decline.^23,24^ This gap indicates the need for the development of multi-analyte panels with rigorous replication and explicit cross-matrix anchoring to clinically meaningful outcomes. Emerging technologies now enable more detailed characterization of periodontal tissues, such as gene sequencing, multi-omics profiling, and advanced bioinformatic analysis.^16,25–29^

Following the Preferred Reporting Items for Systematic reviews and Meta-Analyses extension for Scoping Reviews (PRISMA-ScR) guidelines, this scoping review maps preclinical and clinical evidence on periodontal tissue-derived biomarkers for early AD detection. We sought to (1) construct an AD-specificity pyramid (Tiers 1–4) ordering periodontal biomarkers by proximity to AD pathology, (2) evaluate each candidate's clinical anchoring to brain-relevant endpoints to prioritize biomarkers with the strongest translational potential for prospective validation and chairside deployment, and (3) propose a pragmatic, multi-analyte early-screening panel optimized for potential AD early detection. In simple terms, we ask whether the tissues and fluids that periodontists remove during periodontal treatment could be reused as practical oral samples to test for the risk of AD development instead of simply discarding them.

## Methods

### Protocol and registration

This pre-registered scoping review followed the Arksey and O’Malley framework,^30^ and it is reported in alignment with PRISMA-ScR guidelines.^31^ The protocol has been registered with the Open Science Framework (OSF; Registration *DOI: 10.17605/OSF.IO/EDVU9*, August 20, 2025).

### Eligibility criteria

**Research question**: “What is the current evidence on the feasibility of utilizing periodontal tissues and fluids to identify biomarkers for early AD detection?”

**Population:** Human clinical/translational studies and preclinical *in vivo* animal models relevant to periodontitis/AD.

**Concept:** Biomarkers measured in periodontal tissues and fluids (granulation tissue, gingiva/biopsy, subgingival plaque, and gingival crevicular fluid).

**Context:** Studies that (a) measured biomarkers in periodontal matrices and (b) linked them to AD-relevant endpoints (A/T/N status, cerebrospinal fluid (CSF) amyloid-β (Aβ) positivity) or clinically adjudicated amnestic mild cognitive impairment (aMCI)/AD.

**Inclusion criteria:** (i) Original, peer-reviewed articles in English with full text (through August 2025); (ii) preclinical *in vivo* and/or clinical/translational human studies; and (iii) biomarker measurement in the periodontal tissues and fluids as defined above.

**Exclusion criteria:** (i) non-English or no full text; (ii) non-original articles (reviews, editorials, letters, comments, protocols, patents, and conference abstracts without full text); (iii) *in vitro*-only studies; (iv) studies assessing only saliva or blood, or biomarkers measured only in brain tissue; and (v) studies lacking an explicit link between periodontal biomarkers and AD neurodegeneration.

### Information sources and search strategy

We searched PubMed, Scopus, and Web of Science for studies on periodontal tissue and fluid biomarkers in the context of AD from January 2015 to August 2025**.** The search combined controlled vocabulary and free-text terms for AD dementia and periodontal disease, adapted to each database. Two reviewers (H.Z. and Q.Z.) piloted and refined the strategy; two additional reviewers (Y.O. and L.A.) provided periodontal and neurological input. The full reproducible search strings for each database are provided in Supplemental Table 1. The last search was run on August 20, 2025.

### Study selection

**Figure 1.**
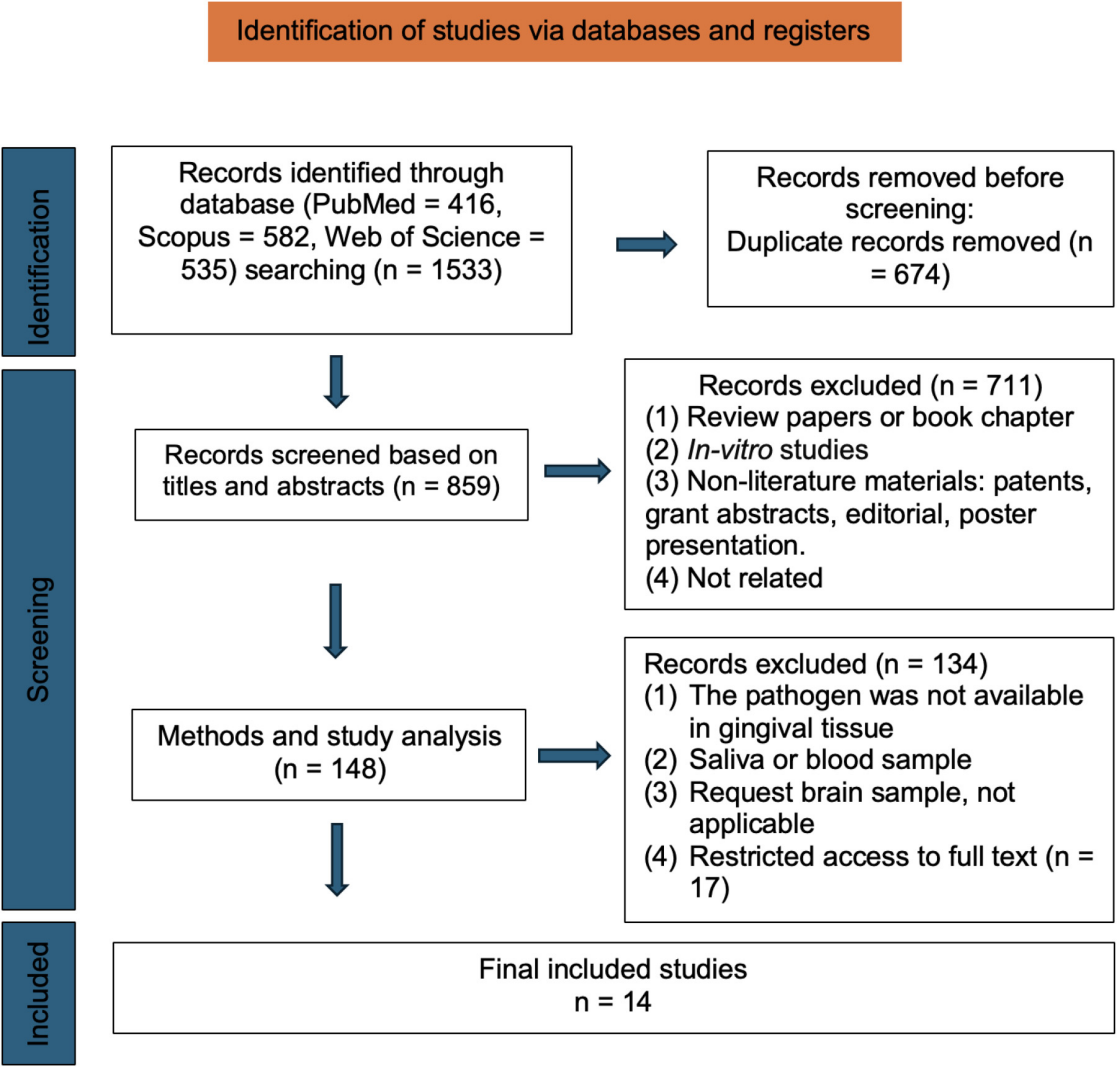
PRISMA-Scr (preferred reporting items for systematic reviews and meta-analyses–extension for scoping reviews) flow diagram of study selection.

One reviewer (Z.C.) manually verified duplicates after automated de-duplication. Four reviewers (two periodontists, H.Z. and L.A., and two neurologists, Q.Z. and Y.O.) independently screened titles/abstracts and then full texts. Disagreements were resolved by consensus; no third-party adjudication was required. The selection process was documented in a PRISMA-ScR flow diagram (Figure 1) with counts provided at each stage.

### Data extraction and charting

**Figure 2.**
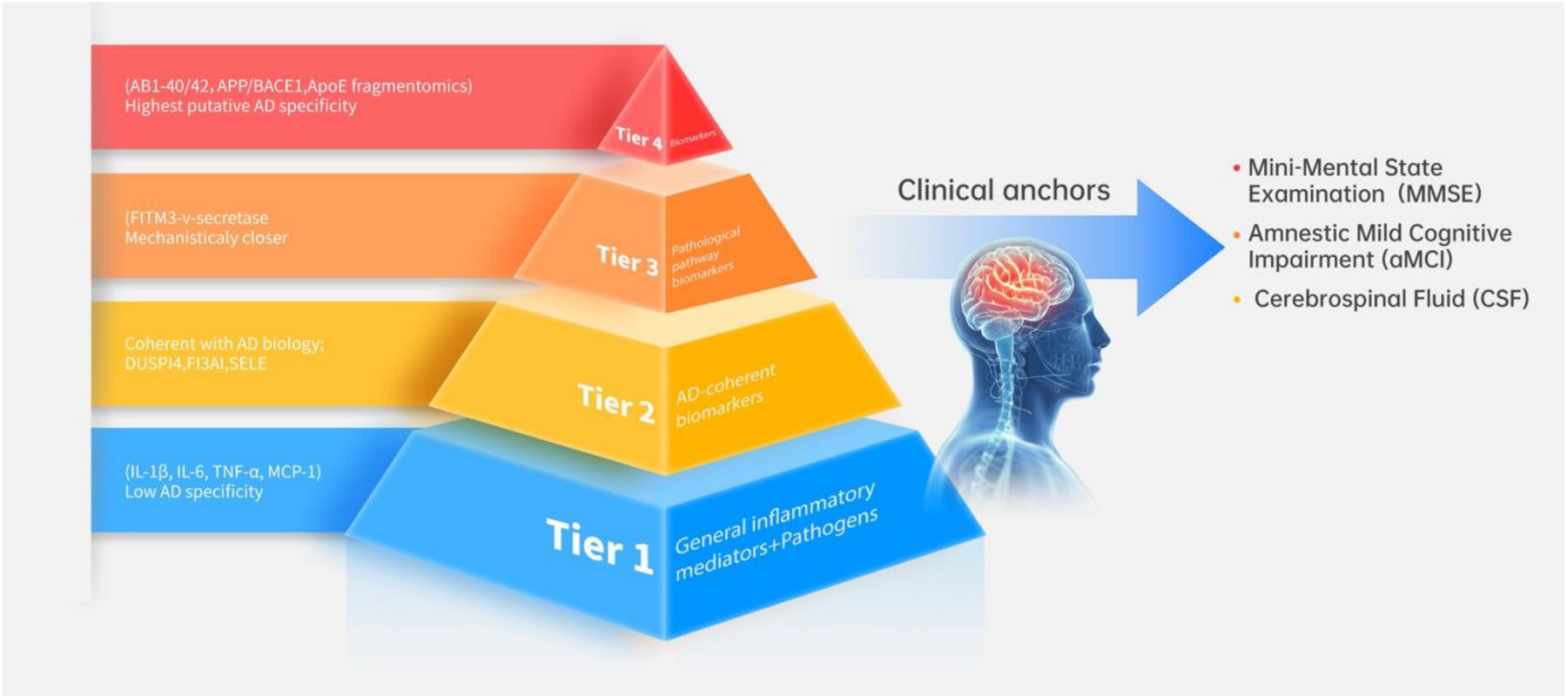
Periodontal-to-Alzheimer's disease biomarker specificity pyramid and clinical anchors.

Two periodontal specialists (H.Z. and L.A.) independently extracted data into a data-charting template (Table 1), including the following key characteristics: authors, year, study design, sample type, biomarkers, and techniques applied. We added two columns: AD specificity tier (Tiers 1–4; as per our specificity pyramid, Figure 2) and clinical anchoring (CSF Aβ positivity, aMCI status/conversion, or clinical diagnosis). Data were managed in a cloud-hosted spreadsheet with version control. The extracted data were then independently reviewed by two reviewers, Q.Z. and Y.O. (consultant neurologists and clinical professors in Neurology). In line with scoping review methodology, no formal risk-of-bias appraisal was undertaken. The findings were synthesized descriptively.

**Table 1.** Characteristics of included studies with specificity tier and clinical anchoring.

Author	Country	Sample type	Study design	Specific biomarker name(s) from gingival tissue	Techniques used	AD specificity	Clinical anchoring
Shen et al.^32^	China	Murine	In vivo	AβPP isoforms (AβPP695, AβPP770); BACE1; Aβ_1–42_	RT-PCR; ELISA	Tier-4	N/A
Na et al.^33^	Korea	Human	Cross-sectional clinical observation	Abundance of fusobacterium, prevotella, saccharibacteria (TM7), Treponema, and Selenomonas in subgingival plaque sample	16S rRNA gene amplicon sequencing	Tier-1	N/A
Kanagasingam et al.^34^	United Kingdom	Human	Ex vivo observational pilot study	Amyloid-β	Transmission Electron Microscopy (TEM), Immunohistochemistry (IHC)	Tier-4	N/A
Franciotti et al.^35^	Italy	Human	Cross-sectional clinical observation	Porphyromonas gingivalis	TaqMan qPCR, ELISA	Tier-1	Diagnosis
Nezu et al.^36^	Japan	Human	Cross-sectional clinical observation	AβPP (amyloid-β A4 precursor protein), NEP (neprilysin)	qRT-PCR, Immunohistochemistry (IHC)	Tier-4 and Tier-3	N/A
Ma et al.^8^	Korea	Murine	In vivo	P. gingivalis-derived extracellular vesicles	ELISA, FITC-Labeling with confocal microscopy, SDS-PAGE densitometry & LC-MS/MS	Tier-3	N/A
Chen et al.^37^	China	Human Datasets	cross-sectional, in silico	MLKL, DCN, IL1β, IL18 gene	Gene Ontology Analysis	Tier-1 and Tier-3	N/A
Wang et al.^38^	China	Murine	In vivo	MCP-1, IL-1β, IL-18	qPCR	Tier-1	N/A
Jin et al.^28^	China	Human	Cross-sectional, in silico	DUSP14, F13A1 and SELE	Microarray gene expression profiling	Tier-2	N/A
Leblhuber et al.^39^	Austria	Human	Cross-sectional clinical observation	Porphyromonas gingivalis; Treponema denticola; Tannerella forsytia	qRT-PCR	Tier-3 and Tier-1	MMSE and CDT
Kamer et al.^40^	USA	Human	Cross-sectional clinical observation	Prevotella oris, P. denticola, Porphyromonas endodontalis, Fretibacterium fastidiosum	16S rRNA gene amplicon sequencing	Tier-1 Tier-4	CSF Ab Positivity
Kong et al.^41^	China	Murine	In vivo	IFITM3	qRT-PCR, Immunohistochemistry (IHC)	Tier-3	N/A
Qiu et al.^42^	China	Human	Cross sectional clinical observation	19 metabolites in GCF and five bacterial species	16S rRNA gene amplicon sequencing; bioinformatic analysis	Tier-3	Clinical Diagnosis
Guo et al.^43^	China	Human	Cross-sectional Clinical observation	*Veillonella parvula and Porphyromonas gingivalis*	16S rRNA gene amplicon sequencing	Tier-1	MMSE Diagnosis

### Synthesis of results

For narrative clarity, the included studies were grouped into three modalities: (1) microbiomes (taxa, co-occurrence, and pathogen genes/virulence/EVs); (2) molecular (proteins, peptides, polysaccharides, extracellular vesicles, and metabolites); and (3) genetic/transcriptomic (gene expression and RNA-seq/microarray). We identified results by biomarker modality and AD-specificity tier (Tiers 1–4) with AD clinical anchors (aMCI or diagnosis). Descriptive landscape figures (country, journal, and year trends) are provided in Supplemental Figure 1–4; the specificity pyramid and clinical anchoring are depicted in Figure 2.

## Results

### Study selection and characteristics

The article selection process is outlined in Figure 1. A total of 859 articles were identified for screening by title/abstract after the removal of duplicates; and 711 texts were excluded for not meeting the inclusion criteria. After the full-text review and article downloading, 14 studies were included in the final analysis. A total of 134 articles were excluded due to (i) no full text available, (ii) saliva- or blood-only sample collection, (iii) brain-tissue-only biomarkers, or (iv) no biomarker measured in gingival/periodontal tissues, subgingival plaque, or gingival crevicular fluid (GCF). Study-level details are provided in Table 1.

Supplemental Figure 1 shows the country distribution of the 14 included studies. Half of the studies were conducted in China (50.0%), and 14.3% of the studies were conducted in Korea. The United States, the United Kingdom, Italy, Japan, and Austria each contributed 7.1% of the publications. Supplemental Figure 2 presents the distribution of sample types among the 14 included studies. Human-derived samples were most frequently used, accounting for 64.3% of the studies. Murine models accounted for 28.6%, whereas publicly available datasets were used in 7%. Supplemental Figure 3 shows the distribution of the 14 included studies across different journals. The publications were distributed across multiple journals, with no single journal dominating this emerging research area. Supplemental Figure 4 shows the annual distribution of the 14 included studies from 2017 to 2025. The earliest relevant publications appeared in 2017 and 2020 (n = 1 each). A modest increase was observed in 2021 (n = 2), followed by a single study in 2022. The number of publications then rose sharply, reaching a peak in both 2023 and 2024 (n = 4 each). In 2025, only one study was identified, as of August. Overall, the data indicate a growing research output in recent years, with a pronounced surge from 2023 to 2024.

### Microbiome-based testing

Na et al. collected subgingival plaques and buccal swabs from AD patients and controls. They reported enrichment in the AD versus control groups in bacteria from the phyla *Fusobacteria, TM7*, and *Spirochaetes*, particularly genera such as *Prevotella, Fusobacterium, Treponema,* and *Selenomonas*.^33^ Specific species enriched in the subgingival plaque of AD patients included *Atopobium rimae*, *Dialister pneumosintes*, *Olsenella* (HMT 807), and TM7 (HMT 348).^33^ Furthermore, they found that in AD patients, bacterial co-occurrence networks were centered on *Prevotella* and were closely linked with *Dialister*. Additionally, genera such as *Olsenella*, *Atopobium*, and *TM7* (HMT348) showed positive associations with *Capnocytophage*.^33^ Leblhuber et al. also reported that approximately 35% of cognitively impaired patients harbored key periodontal pathogens, specifically *P. gingivalis*, *T. denticola*, *and T. forsythia*.^39^ These findings indicate a distinct bacterial profile in AD patients compared with controls; however, brain/CSF linkage or clinical anchoring is lacking, and these bacteria constitute a Tier-1 (non-specific) dysbiosis/context rather than representing AD-specific biomarkers. Kamer et al. reported that the subgingival microbial profile is altered in AD pathology and even in preclinical AD. In addition, the genus level dysbiosis index, defined as (*Treponema/Porphyromonas/Tannerella*) divided by (*Rothia/Corynebacterium*), is higher in CSF Aβ–positive patients than in Aβ-negative patients.^40^ Species-level clustering revealed a periodontal pathogenic cluster (e.g., *Prevotella oris*, *P. denticola*, *Porphyromonas endodontalis*, and *Fretibacterium fastidiosum*) associated with lower CSF Aβ_42_ and markedly higher odds of Aβ-positivity, with no association observed for CSF P-tau.^40^ Guo et al. observed oral microbiome shifts with enrichment of *Veillonella parvula* and *Porphyromonas gingivalis* in an AD cohort, nominating these as candidate discriminatory taxa.^43^

### Molecular-based biomarkers

Using IHC and TEM, Kanagasingam et al. detected soluble and insoluble Aβ assemblies within the extracellular polymeric substance of native periodontal/endodontic biofilms—constituting Tier-4 core pathology biomarkers in periodontal tissues and fluids.^34^ In a mouse model, Shen et al. demonstrated that *Porphyromonas gingivalis* (Pg*)*–induced periodontitis promoted abnormal amyloid-β protein precursor (AβPP) processing and increased Aβ metabolites in gingival tissues and the peripheral circulation.^32^ Specifically, the periodontal tissue of Pg-infected mice showed upregulated expression of AβPP isoforms (AβPP695 and AβPP770) and the AβPP-cleaving enzyme BACE1, accompanied by elevated levels of Aβ_1–40/42_ in the GCF and plasma and paradoxical downregulation of BACE1 mRNA in the brain cortex.^32^ AβPP/BACE1 dynamics and Aβ species are core AD-related processes; these biomarkers are classified as Tier 4 (core pathology biomarkers) and are demonstrated in periodontal tissues and fluids.^44^ Patel et al. revealed that gingipain proteases (RgpA, RgpB, and Kgp) abundantly secreted by *P. gingivalis* can cleave apolipoprotein E (ApoE), central to AD genetic risk, into fragments.^45–47^ ApoE is a core AD molecule, and the evidence pertains to a pathway-proximal proteolytic mechanism, classified as a Tier 4/3 interface as per our specificity outcomes (core pathology anchor and pathway mechanism).^46,47^ Ma et al. found that *P. gingivalis*-derived extracellular vesicles (pEVs) were sufficient to induce neuroinflammatory changes and cognitive impairment in their experimental model even in the absence of living bacteria, indicating that pEVs alone can contribute to AD-related pathology.^8^ Thus, pEVs are a potential mechanistic link proximal to the AD-relevant pathway that do not constitute core AD-specific biomarkers (e.g., Aβ species, AβPP/BACE1 dynamics, and ApoE); hence, pEVs were categorized as Tier 3 (pathway proximal signals). Qiu et al. extended the investigation to humans with aMCI, integrating GCF metabolic profiles with microbial analyses.^42^ They identified a combined panel of 19 GCF metabolites and five bacterial species correlated with Montreal Cognitive Assessment (MoCA)/Mini-Mental State Examination (MMSE) and periodontal indices that could serve as a potential biomarker profile for early AD detection with clinical anchoring.^42^ The species-level dysbiosis and GCF metabolite analysis in this study were classified as Tier 1 (pathogens) with Tier-3 adjacency (metabolites proximal to host–microbe pathway), given the lack of direct alignment to the Tier 4 anchor (core AD pathology).

### Genetic and transcriptomic biomarkers

Franciotti et al. quantified *P. gingivalis* from tongue-dorsum swabs and found higher bacterial loads in neurodegenerative (ND: including AD) versus non-ND and healthy groups. This finding reflects Tier-1 (pathogens) background dysbiosis and is anchored only to broad clinical diagnosis (no A/T/N).^35^ Nezu et al. showed increased AβPP and neprilysin (NEP) mRNA in periodontitis-affected gingiva with IHC localization (AβPP in inflammatory cells; NEP in spindle-shaped cells), classifying AβPP as Tier 4 (core pathology anchor) and NEP as Tier 3 (pathological proximal pathway), without clinical anchoring.^36^ In a *P. gingivalis* periodontitis mouse model, Kong et al. reported upregulated IFITM3 in periodontal tissues and fluids, increased brain Aβ, and cognitive deficits—evidence of a Tier-3 IFITM3–γ-secretase axis with Tier-4 manifestations *in vivo*, but no human anchoring.^41^ In addition to experimental studies, some researchers mined publicly available transcriptomic datasets to explore connections between periodontitis and AD via bioinformatic analysis.^28,37^ Using public transcriptomes, Chen et al. identified periodontal pathology–AD “crosstalk” genes (e.g., IL1B, IL18, DCN, and MLKL) indicative of immune/PANoptosis links; these remain within the Tier 1–3 biomarker pyramid without periodontal biomarker validation or AD anchoring.^37^ Similarly, Jin et al. found 364 crosstalk genes among the differentially expressed genes between AD and periodontitis. They further narrowed these genes into a three-gene signature (DUSP14, F13A1, and SELE) that represents a robust immune-related molecular profile common to both diseases, yet lacks clinical or pre-clinical validation and anchoring.^28^

## Discussion

By mapping biomarkers relevant to AD that can be obtained from periodontal tissues and fluids, our review contributes to current research on novel biomarkers, mechanisms, and translational pathways for early AD detection. Saliva and blood often exhibit low analyte abundance, cross-reactivity, and systemic variability, which can confound associations—particularly for circulating pathogen antigens or antibodies.^48,49^ In contrast, granulation tissue, GCF, and subgingival plaque are routinely obtained during standard care. These tissues and fluids concentrate local biomarkers and enable cellular and spatial assays that are rarely feasible in saliva.^15–19^ We organized biomarkers into a specificity pyramid (Figure 2). We classified general inflammatory mediators not specific to AD pathology, such as cytokines (IL-1β, IL-6, TNF-α, MCP-1) and microbiome pathogen composition (*Porphyromonas*, *Treponema*, *Tannerella*, *Prevotella*; dysbiosis indices), as Tier 1.^33,35,37–39^ Some immune crosstalk genes (e.g., DUSP14, F13A1, and SELE) indicated convergent immune biology but lacked *in vivo* validation against brain endpoints; thus, they were placed in Tier 2.^28^ When biomarkers were directly involved in pathological mechanisms but were not AD-specific biomarkers in periodontal tissues and fluids, they were categorized as Tier 3. For example, IFITM3 upregulation linked interferon signaling to γ-secretase modulation and increased Aβ production.^23,41^ *P. gingivalis*–derived extracellular vesicles transmit virulence cargo from the gingiva to the trigeminal/hippocampal structures and elicit hippocampal inflammatory signals.^8^ Gingipain-mediated proteolysis of host substrates, such as ApoE, supports the pathological pathway; however, human brain-anchored validation is required. These pathways are biologically proximate to amyloidogenic/neuroinflammatory pathways but have not yet been anchored to brain biomarkers in humans. In some studies, periodontal tissue and fluid have shown Aβ assemblies and AβPP/BACE1 upregulation, with Aβ_1–40/42_ detected, representing core-pathology biomarkers in periodontal tissues and fluids.^32,34,36^ In contrast, tau/phospho-tau has not been reliably detected in gingival tissues within the studies included in this review. The biomarkers included in the studies span one or two tiers, but none span the full pyramid from Tier 1 to Tier 4 within a single design. Five studies used clinical anchors, such as clinical diagnosis, MMSE, CDT, or CSF Aβ positivity, with CSF Aβ positivity as the applicable brain-relevant biological anchor, making it feasible for early AD detection.^35,39,40,42,43^ MMSE and CDT are useful clinical tools for cognitive screening and severity grading in neurology co-management, but they do not constitute a biological anchor attributable to dentistry.

A pragmatic strategy is to assemble a multitier panel that integrates complementary biomarkers across the specificity pyramid (Figure 2). Tier 1 features, such as general inflammatory mediators (e.g., IL-1β, IL-6, TNF-α, and MCP-1) and the presence or imbalance of local periodontal pathogens, can be used to capture contextual inflammation and the microbial burden. Tier 2 AD-coherent immune biomarkers (e.g., DUSP14, F13A1, and SELE) reflect innate/vascular activation consistent with AD biology. Tier 3 pathological pathway biomarkers (e.g., IFITM3 or gingipain/pEV-related host responses) can then be layered in; these are mechanistically close to amyloidogenic/neuroinflammatory cascades. At the apex, Tier 4 core pathology anchors measurable in periodontal matrices (e.g., Aβ species or AβPP/BACE1 dynamics) can be incorporated. Finally, the composite score can be interpreted against a feasible clinical anchor, preferably CSF Aβ positivity (and, where available, clinically adjudicated aMCI/diagnosis), to enhance translational validity for early AD detection.

To maximize translational feasibility at the chairside, we propose an early-detection panel deliberately limited to qPCR/qRT-PCR–based assays across tiers. This choice is supported by our 14 included studies, which show that qPCR/qRT-PCR is a widely available platform capable of covering all selected biomarkers from Tier 1 to Tier 4. Periodontal granulation tissue and standardized subgingival plaque can be collected via a curette during periodontal treatment.

Collected granulation tissue, GCF, and subgingival plaque can be processed on a unified qPCR/qRT-PCR platform to span the proposed specificity tiers. Tier 1 (pathogen): targeted qPCR on plaque DNA for *P. gingivalis*, *Treponema* spp., and *Tannerella forsythia* (±*Prevotella*), reported as ΔCt values or a dysbiosis index.^33,35,39,43^ Tier 2 (AD-coherent immune biomarker): triplex qRT-PCR of DUSP14, F13A1, and SELE using granulation-tissue RNA, aggregated into a normalized expression score(28). Tier 3 (pathological pathway): qRT-PCR quantification of IFITM3 (interferon–γ-secretase axis) in granulation tissue, expressed as standardized fold-change.^41^ Tier 4 (core-pathology adjacency): qRT-PCR–based AβPP/BACE1 ratio derived from granulation-tissue RNA as an RNA-level surrogate of amyloidogenic processing.^32^ This unified workflow minimizes assay complexity while capturing contextual microbial load, AD-coherent immune activity, pathway proximity, and core pathology dynamics in periodontal matrices, suitable for early detection. Where feasible, periodontists could implement brief cognitive screening or referral pathways, while neurologists/geriatricians could incorporate periodontal assessment into risk management.^50,51^ Our findings encourage a more interdisciplinary approach to AD risk screening.

### Limitations

The evidence base is small (14 studies) and methodologically heterogeneous across species (animal versus human), origin of collection, and assay platforms. We did not perform a formal risk-of-bias or certainty appraisal, consistent with the scoping methodology; the findings are hypothesis-generating. Most human evidence is cross-sectional, limiting inference regarding whether periodontal biomarkers predict AD outcomes. Despite comprehensive searching, language and database restrictions, along with unavailable full texts, may have introduced selection and publication bias.

### Conclusion

Periodontal tissues and fluids constitute a practical, high-signal “oral window” for AD-relevant biology that can be sampled during routine care. To translate this promise, the field now needs prospective, pre-registered, and protocol-standardized studies that quantify multitier biomarkers across matrices (granulation tissue, GCF, and plaque) and anchor them to brain-relevant endpoints (A/T/N metrics and CSF Aβ positivity) and adjudicated clinical outcomes (aMCI and conversion). Harmonized pre-analytics and qPCR/qRT-PCR–centered workflows should enable reproducible, chairside panels spanning dysbiosis, AD-coherent immune transcripts, pathway-proximal signals, and AβPP/BACE1 dynamics. Such evidence will support integrated oral–brain screening pathways within periodontal practice and co-managed neurology care.

## Supplemental Material

sj-docx-1-alr-10.1177_25424823261421629 - Supplemental material for From routine periodontal therapy to Alzheimer's disease early detection: A scoping review Supplemental material, sj-docx-1-alr-10.1177_25424823261421629 for From routine periodontal therapy to Alzheimer's disease early detection: A scoping review by Qiang Zhang, Lina Almanie, Yi Ouyang, Zihao Cheng and Hengjia Zhang in Journal of Alzheimer's Disease Reports
