# Supplementary material for: From routine periodontal therapy to Alzheimer's disease early detection: A scoping review
Source: J Alzheimers Dis Rep. 2026 Feb 4;10:25424823261421629. doi: 10.1177/25424823261421629 (PMC13039049; doi:10.1177/25424823261421629)
Supplement: sj-docx-1-alr-10.1177_25424823261421629 - Supplemental material for From routine periodontal therapy to Alzheimer's disease early detection: A scoping review [file sj-docx-1-alr-10.1177_25424823261421629.docx]

**Supplemental Material**

**From routine periodontal therapy to Alzheimer’s disease early detection: A scoping review**

**Supplemental Table 1.** Search strategy for each database.

| **Database** | **Search Strategy** |
| --- | --- |
| PubMed | <https://pubmed.ncbi.nlm.nih.gov/?term=%28%28Alzheimer%5Btiab%5D+OR+%22Alzheimer+Disease%22%5BMeSH%5D+OR+dementia%5Btiab%5D%29AND+%28periodontal%5Btiab%5D+OR+periodontitis%5Btiab%5D%29%29&filter=datesearch.y_10&filter=simsearch3.fft&filter=lang.english&filter=hum_ani.humans&filter=hum_ani.animal>  ((Alzheimer[tiab] OR "Alzheimer Disease"[MeSH] OR dementia[tiab])AND(periodontal[tiab] OR periodontitis[tiab])) |
| Scopus | TITLE-ABS-KEY ( ( Alzheimer* OR dementia ) AND ( periodontal OR periodontitis ) ) AND PUBYEAR > 2014 AND PUBYEAR < 2026 AND ( LIMIT-TO ( LANGUAGE , "English" ) ) AND ( LIMIT-TO ( DOCTYPE , "ar" ) ) |
| Web of Science | <https://www.webofscience.com/wos/alldb/summary/675412ba-4948-4acd-aaa0-2d83ca93080a-0172d09c58/relevance/1>  TS=(Alzheimer OR dementia) AND TS=(periodontal OR periodontitis) |

**Supplemental Figure 1.** Distribution of publications by countries.

**Supplemental Figure 3.** Distribution of sample types across the included studies.

**Supplemental Figure 4.** Distribution of included studies across different journals.

**Supplemental Figure 5.** Annual publication trend of included studies (2017–2025).
